# Dehalogenation reactions between halide salts and phosphate compounds

**DOI:** 10.3389/fchem.2022.976781

**Published:** 2022-09-07

**Authors:** Brian J. Riley, Saehwa Chong

**Affiliations:** Pacific Northwest National Laboratory, Richland, WA, United States

**Keywords:** dehalogenation, hydrogen halides, ammonium halides, nitrogen trihalides, ammonium triiodide, molten salt reactors, electrochemical reprocessing

## Abstract

Reactions between phosphoric acid [H_3_PO_4_] or ammonium hydrogen phosphates [i.e., NH_4_H_2_PO_4_, (NH_4_)_2_HPO_4_] and halide salts can be used to dehalogenate (remove halides from) salt-based waste streams, where the process of removing halides yields products that have more efficient disposal pathways for repository storage. In this context, the term efficiency is defined as higher waste loadings and simplified immobilization processes with potential for recycle of certain salt components (e.g., ^37^Cl as H^37^Cl or NH_4_^37^Cl). The main streams identified for these processes are nuclear wastes generated during electrochemical reprocessing of used nuclear fuel as well as used halide salts from molten salt reactor operation. The potential byproducts of these reactions are fairly consistent across the range of halide species (i.e., F, Cl, Br, I) where the most common are hydrogen halides [e.g., HCl_(g)_] or ammonium halides (e.g., NH_4_Cl). However, trihalide compounds (e.g., NCl_3_), nitrogen triiodide ammine adducts [NI_3_·(NH_3_)_*x*_], and ammonium triiodide (NH_4_I_3_) are also possible. Several of these byproducts (i.e., NCl_3_, NBr_3_, NI_3_, and NH_4_I_3_) are shock-sensitive contact explosives so their production in these processes must be tracked and carefully controlled, which includes methods of immediate neutralization upon production such as direct transport to a caustic scrubber for dissolution. Several benefits arise from utilizing H_3_PO_4_ as the phosphate additive during dehalogenation reactions for making iron phosphate waste forms including more oxidized iron (higher Fe^3+^:Fe^2+^ ratios), higher chemical durabilities, and the avoidance of trihalides, but the byproducts are hydrogen halides, which are corrosive and require special handling.

## Introduction

Salt-based nuclear wastes can be generated through electrochemical reprocessing (pyroprocessing) or operation of molten salt reactors (MSRs). If the radionuclide-containing salt wastes cannot be directly disposed in a nuclear waste repository, it is possible that these wastes can be treated prior to disposal to improve the available options for waste form production (Riley, 2020). One of these treatment processes includes dehalogenation of the salt where the halides are removed and 1) recovered and recycled, 2) immobilized in a different form, or 3) potentially discarded. The primary goal of partitioning the wastes is to find more suitable and efficient waste forms for the different waste constituents since halide solubilities in traditional borosilicate glass nuclear waste forms are very low and the retentions of halides during melting to create glassy waste forms are also low (Hrma, 2010; Riley et al., 2012; Riley et al., 2014). Secondary goals include the benefit of recovering valuable isotopes like ^37^Cl for applications like MSRs. While both ^35^Cl and ^37^Cl are stable isotopes, the motivation for using ^37^Cl-enriched salts for MSRs are 1) to prevent neutron activation of natural ^35^Cl to the long-lived radioisotope of ^36^Cl (*t*_1/2_ = 3.01 × 10^5^ years) and 2) to decrease parasitic neutron absorption (McFarlane et al., 2019). Having long-lived radioisotopes in nuclear wastes drives repository dose calculations so they should be minimized, if possible, to save on disposal costs and potential environmental impacts. Since radioiodine is also an issue from a repository dose standpoint due to long-lived ^129^I (*t*_1/2_ = 1.57 × 10^7^ years), selective removal of iodine from these salts and maximizing iodine loading in a high efficiency waste form is also desired. Bromine is a fission product present in nuclear waste streams (Riley et al., 2018) and, unlike the other halogens, is a liquid at room temperature with a boiling temperature of 58.8°C (Lide 2007-2008). Following dehalogenation, immobilization of the fission product oxides could be realized in a phosphate waste form including phosphate glass (e.g., iron phosphate, iron aluminophosphate, aluminophosphate) (Day et al., 1998; Siemer, 2013a; Siemer, 2013b; Day and Ray, 2013; Bai et al., 2021) and/or phosphate-based crystalline matrices (e.g., monazite) (McCarthy et al., 1978; Boatner et al., 1980).

## Dehalogenation reactions and byproducts

Demonstrated methods for dehalogenating chloride-based salt wastes include reactions between chloride-based salts with ammonium phosphates [e.g., NH_4_H_2_PO_4_ and (NH_4_)_2_HPO_4_] shown in Eqs 1, 2 (Donze et al., 2000; Donze et al., 2001; Bekaert et al., 2006; Riley et al., 2020), reactions with H_3_PO_4_ such as that shown in Eq. 3 (Lavrinovich et al., 2003; Park et al., 2007a; Park et al., 2007b; Park et al., 2008; Park et al., 2011; Siemer, 2012; Lee et al., 2019), reactions with hydrogen-based zeolites like ultrastable H-Y zeolite shown in Eq. 4 (Wasnik et al., 2019a; Wasnik et al., 2019b), or through high-temperature reactions with steam to produce metal oxides shown in Eq. 5 (Sato et al., 2002). A third ammonium phosphate material, (NH_4_)_3_PO_4_, will be mentioned here for completion purposes, but it is quite unstable; however, it could potentially be adapted for these types of processes.

$$2NH_{4}H_{2}PO_{4}+2\;\mathrm{NaCl}\to 2\;NH_{4}\mathrm{Cl}+2\;H_{2}O_{\left(g\right)}+Na_{2}O\cdot P_{2}O_{5}$$ (1)

$$2\;{\left(NH_{4}\right)}_{2}\mathrm{HP}O_{4}+4\;\mathrm{NaCl}\to 4\;NH_{4}\mathrm{Cl}+H_{2}O_{\left(g\right)}+{\left(Na_{2}O\right)}_{2}\cdot P_{2}O_{5}$$ (2)

$$2\;H_{3}PO_{4}+4\;\mathrm{NaCl}\to 4\;\mathrm{HC}l_{\left(g\right)}+H_{2}O_{\left(g\right)}+{\left(Na_{2}O\right)}_{2}\cdot P_{2}O_{5}$$ (3)

$$H{\left(\mathrm{Si}O_{2}\right)}_{2.6}\left(\mathrm{Al}O_{2}\right)+\mathrm{NaCl}\to \mathrm{Na}{\left(\mathrm{Si}O_{2}\right)}_{2.6}\left(\mathrm{Al}O_{2}\right)+\mathrm{HC}l_{\left(g\right)}$$ (4)

$$2\;\mathrm{NaCl}+H_{2}O_{\left(g\right)}\to Na_{2}O+2\;\mathrm{HCl}$$ (5)

**FIGURE 1:**
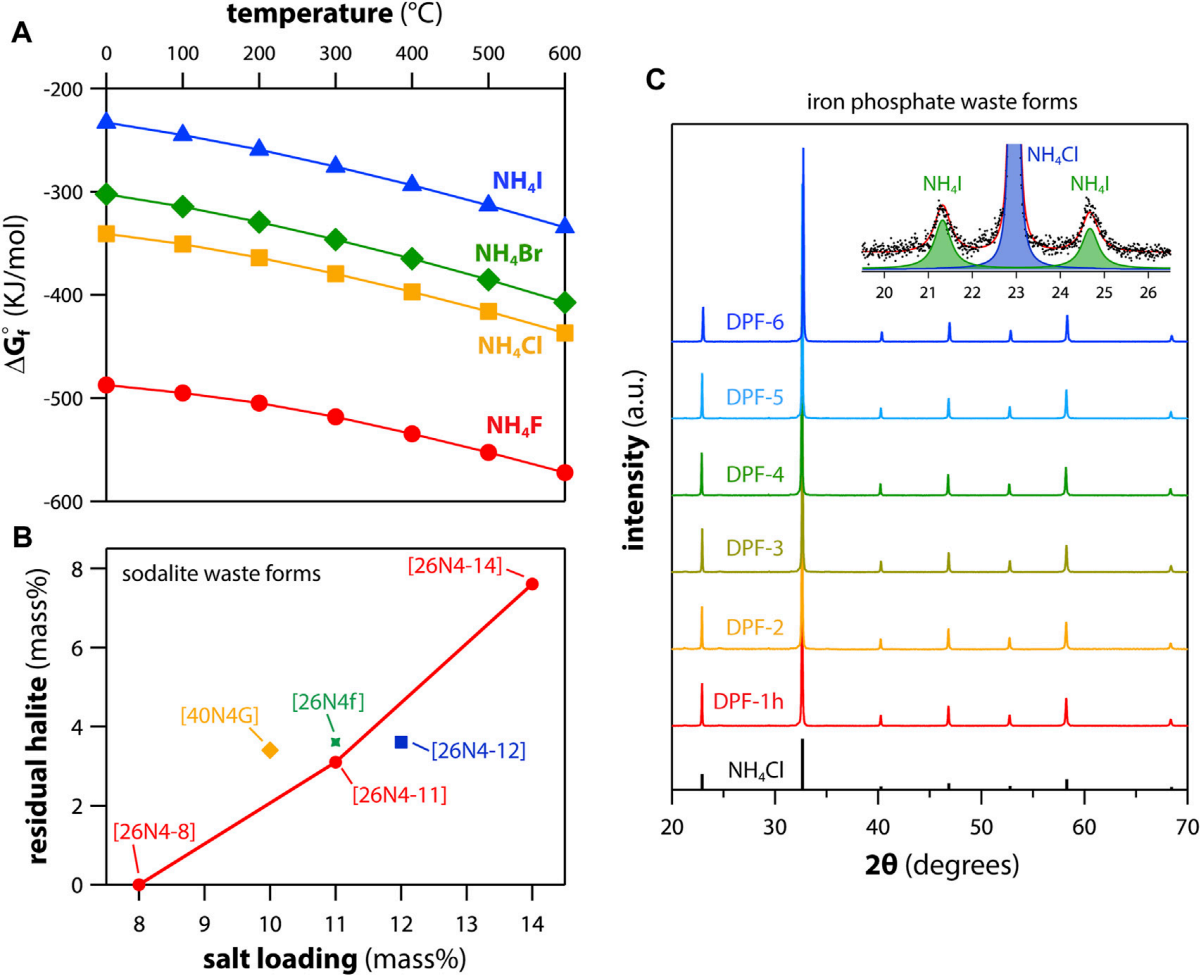
**(A)** Gibbs free energies of formation for various ammonium halide salts (Riley et al., 2020). **(B)** Residual halite (NaCl) mass, based on X-ray diffraction data, as a function of salt loading used to make glass-bonded sodalite waste forms showing that residual salts are observed when the salt fractions are too high (>8 mass%) due to crystal stoichiometry limits (Riley et al., 2017). **(C)** Powder X-ray diffraction data showing the majority phase of NH_4_Cl with a minor NH_4_I phase in solid condensates recovered after a reaction between NH_4_H_2_PO_4_ and a Cl/I-containing salt simulant at temperatures up to 600°C in an alumina crucible (Riley et al., 2020). Parts **(A,C)** of this figure were modified from the originals by Riley et al. (2020) and were reprinted with permission. Copyright Elsevier (2020). Part **(B)** was modified from the original by Riley et al. (2017) and reprinted with permission. Copyright Elsevier (2017).

Dehalogenation processes are useful for waste form production of chloride-based salt wastes because it allows for much higher waste loadings in the final waste form for the remaining fission products because the halides no longer limit this loading capacity as it does with other salt waste form options like crystalline matrices with physical halide limits such as chlorosodalite [e.g., Na_8_(AlSiO_4_)_6_Cl] (Vance et al., 2012) and chlorapatite [e.g., Ca_5_(PO_4_)_3_Cl] (Vance et al., 2012) due to crystal chemistry stoichiometries (Figure 1B). When the salt loading limits are exceeded for crystalline-based halide-host matrices, residual salts can be observed (Figure 1B), which are not a chemically durable or stable form for long-term disposal.

Fluoride salts can be processed using phosphate precursors as well. Wang et al. (2004) demonstrated that NH_4_H_2_PO_4_ can be used to defluorinate LiF salt with a byproduct of NH_4_F through Eq. 6 or produce NH_4_F through the reaction between HF_(g)_ and NH_3(g)_ as shown in Eq. 7. It is likely that these types of reversible reactions could take place at different times in the same system depending on the experimental conditions. Regarding Eq. 7, both hydrogen halides (e.g., HCl) and NH_3(g)_ can be found as byproducts of these reactions as well as in the decomposition reactions of the phosphate reagents. However, to the knowledge of the authors, no such studies have been performed starting from pure iodine-containing salts. In a study by Xiang et al. (2019), NH_4_H_2_PO_4_, Cs_2_CO_3_, SrCO_3_, PbBr_2_, and NaBr were reacted together resulting in a 45P_2_O_5_-20PbBr_2_-10NaBr-13Cs_2_O-12SrO glass containing CsPbBr_3_ crystals after a mechanical stress was applied. This study provides evidence of Br retention after heating NH_4_H_2_PO_4_ and Br-containing compounds to 680°C in air.

$$2\;NH_{4}H_{2}PO_{4}+2\;\mathrm{LiF}\to 2\;NH_{4}F+2\;H_{2}O_{\left(g\right)}+Li_{2}O\cdot P_{2}O_{5}$$ (6)

$$HF_{\left(g\right)}+NH_{3\left(g\right)}\to NH_{4}F$$ (7)

These types of reactions can be studied in real-time using characterization techniques like differential scanning calorimetry (DSC; i.e., phase change temperatures, heats of reaction), thermogravimetric analysis (TGA; i.e., mass loss over a range of changing temperatures and/or times), evolved gas analysis (EGA; i.e., identification of off-gas species from the reactions) with an attached gas chromatograph and mass spectrometer, or hot stage X-ray diffraction. In a recent study (Riley et al., 2021a), EGA was utilized to study NH_4_Cl decomposition as well as monitor the reactions between NH_4_H_2_PO_4_ and KCl. This study showed similar byproducts of NH_3_, HCl, and H_2_O for both of these experiments at temperatures as low as ∼200–300°C providing evidence that heat treatment temperatures, heating rates, and temperature dwell times are important parameters for preventing decomposition of the byproducts if ammonium halide salt products are desired [Eq. 8].

$$NH_{4}Cl_{\left(s\right)}\to NH_{3\left(g\right)}+\mathrm{HC}l_{\left(g\right)}$$ (8)

**FIGURE 2:**
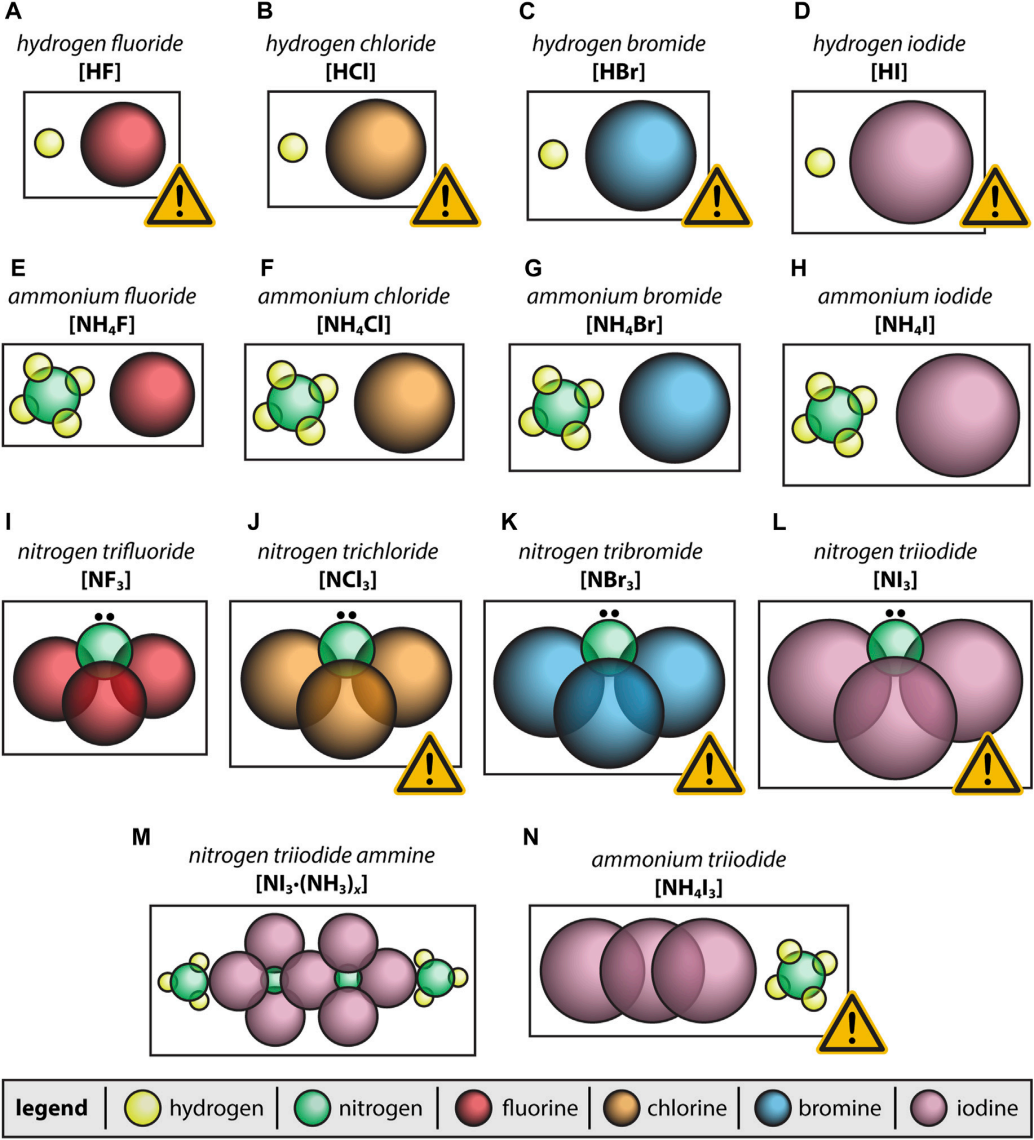
Potential halogen-based byproducts generated during dehalogenation processes including **(A–D)** hydrogen halides; **(E–H)** ammonium halides; **(I–L)** nitrogen trihalides; **(M)** NI_3_·(NH_3_)_*x*_ ammine adduct where *x* = 1, 2, 3, 5, or 12; and **(N)** NH_4_I_3_. The yellow triangles (!) for some species indicate additional hazards that are described in the text in more detail.

Thermodynamic calculations performed using HSC Chemistry show that the ammonium halides should form spontaneously based on negative Gibbs free energies of formation (Δ*G*_f_°) across the temperature range of 0 ≤ *T* ≤ 600°C as shown in Figure 1A (Riley et al., 2020). Also, the Δ*G*_f_° values show that the formation preference of the ammonium halides is in the order of NH_4_F → NH_4_Cl → NH_4_Br → NH_4_I with the more favorable reactions being the lighter ammonium-halide complexes (Figure 1A and Figures 2E–H). In the presence of salt simulants with both chlorine and iodine, both NH_4_Cl and NH_4_I were observed in the solid condensates after reactions with NH_4_H_2_PO_4_ at temperatures up to 600°C (Figure 1C) (Riley et al., 2020). However, what these calculations did not show was the formation of other, unwanted potential byproducts when nitrogen-containing reactants are used to dehalogenate some of these salts.

In reactions involving nitrogen-containing compounds and halide vapors, complexes such as nitrogen trihalides (i.e., NF_3_, NCl_3_, NBr_3_, and NI_3_) can form as well as ammonium triiodide (e.g., NH_4_I_3_) or ammines (adducts) of ammonia nitrogen triiodide [NI_3_·(NH_3_)_*x*_] where *x* = 1, 2, 3, 5, or 12 according to Matyáš and Pachman (2013) (Figure 2M). The pure hydrogen halide compounds HF, HCl, HBr, and HI are all colorless gases at room temperature with different boiling temperatures (*T*_b_s) of 19.5°C, −85°C, −66.8°C, and −35.4°C, respectively (Lide 2007-2008; Matyáš and Pachman, 2013) (see Figures 2A–D). The ammonium halide salts are all white in appearance and some can decompose when heated into NH_3(g)_ and hydrogen halides [e.g., Eq. 8]. For instance, NH_4_F decomposes at 100°C, NH_4_Cl decomposes at 338°C, NH_4_Br boils at 452°C, and NH_4_I sublimes at 235°C (Lide 2007-2008; Matyáš and Pachman, 2013). The nitrogen trihalide compounds NF_3_, NCl_3_, and NI_3_ all behave differently with *T*_b_s of −129.1°C, 71°C, and −20°C (sublimation temperature) and have different appearances of a colorless gas, a yellow oily liquid, and a dark solid, respectively (Lide 2007-2008; Matyáš and Pachman, 2013) (see Figures 2I, J, L). The compound NBr_3_ is a deep red solid, and the *T*_b_ could not be found reported in the literature but it is known to be explosive at temperatures as low as −100°C even under slight disturbances (Jander, 1976) (see Figure 2K), likely making the *T*_b_ determination difficult. The ammine compounds of NI_3_·(NH_3_)_*x*_ are black-colored crystals and this is the expected appearance of NH_4_I_3_ as well (Fedoroff et al., 1975) (see Figure 2N).

The primary concern with NCl_3_, NBr_3_, NI_3_, and NH_4_I_3_ is that they are known contact explosives (Matyáš and Pachman, 2013); NF_3_ is not a contact explosive but it is both a toxic gas and greenhouse gas with high global warming potential (Tsai, 2008). Contact explosives are highly unstable materials that can react or explode violently when exposed even to very small amounts of external energy (e.g., gentle contact, sound, α particles, light, spark discharge, mild heating) or strong light and can do so in the absence of oxygen (e.g., in an inert glovebag, glovebox, or hot cell) (Henderson, 1922; Meerkämper, 1954; Bowden, 1958; Fedoroff et al., 1975; Matyáš and Pachman, 2013). These high-energy reactions can proceed to produce diatomic halide gases [e.g., I_2(g)_] along with other byproducts documented through experimentation (or proposed) in Eqs 9–11 (Holleman and Wiberg, 2001) and are often used in chemistry demonstrations for students.

$$2\;NI_{3\left(s\right)}\to N_{2\left(g\right)}+3\;I_{2\left(g\right)}$$ (9)

$$8\;\left(NI_{3}\cdot NH_{3}\right)\to 5\;N_{2\left(g\right)}+6\;NH_{4}I+9\;I_{2\left(g\right)}$$ (10)

$$NH_{4}I_{3\left(s\right)}\to NH_{4}I+I_{2\left(g\right)}$$ (11)

Alternatively, both NH_4_I_3_ and NI_3_ can be neutralized chemically through reactions with high-pH solutions such as those present within a caustic scrubber whereby the complexes are dissolved and dissociate into more stable (less dangerous) species. Aqueous caustic scrubbers are often used in nuclear applications to neutralize acidic species and an alternative to this approach with similar capabilities would be a nonaqueous molten hydroxide scrubber (Haefner and Tranter, 2007; Riley et al., 2016; Riley et al., 2019; Andrews et al., 2021; Bollinger et al., 2022). These acids include the hydrogen halide acids (i.e., HF, HCl, HBr, and HI; Figures 2A–D), which are likely byproducts when H_3_PO_4_ or ammonium phosphates are present, all of which can be neutralized through the hydroxide ions present within caustic scrubbers. However, it is possible that other acids will be present in these streams as well depending on the application (e.g., HNO_3_), which would also be neutralized by the caustic scrubber or molten hydroxide scrubber, which is a secondary benefit.

Despite the volatile and solid-condensable byproducts [e.g., hydrogen halide gases, H_2_O_(g)_, N_2(g)_, NH_3(g)_, ammonium halides] and the initial salt chemistry, if these reactions are performed in air, it is likely that the material remaining in the crucible after dehalogenation of halide salts with phosphate precursors [i.e., H_3_PO_4_, NH_4_H_2_PO_4_, (NH_4_)_2_HPO_4_] would be fairly consistent in composition no matter which phosphate reactant is used where the salt cations are converted from halides to oxides within a P_2_O_5_ matrix [Eqs 1–3, 5 above]. This is one of the more important benefits of dehalogenation as it greatly simplifies the next steps required to fully immobilize the remaining product, which can be reacted with glass-forming chemicals (e.g., Fe_2_O_3_) to produce a chemically durable waste form for disposal in a nuclear waste repository (Park et al., 2007a; Park et al., 2007b; Park et al., 2008; Park et al., 2011; Siemer, 2012; Ebert et al., 2018; Ebert and Fortner, 2019a; Ebert and Fortner, 2019b; Lee et al., 2019; Ebert and Fortner, 2020; Stariha and Ebert, 2020; Riley et al., 2021b; Stariha and Ebert, 2021).

The fates of the halides following dehalogenation need to be considered. If halide recycle is desired such as the recovery of valuable ^37^Cl from MSR-based wastes, capture as HCl or NH_4_Cl should provide multiple pathways for reuse. One such option for ^37^Cl recycle is to use NH_4_Cl to convert U^0^ to UCl_3_ that potentially could be returned to MSRs as a fuel source or as an oxidant for electrochemical reprocessing (Herrmann, 2017; Frank et al., 2018; Riley et al., 2020). For Cl, Br, and I, if these are captured in caustic scrubbers, the products can likely be immobilized directly in a halide-specific waste form like sodalite or apatite starting from these halide-containing solutions and reacting them with reagents (e.g., zeolites) at room temperature and atmospheric pressure or at elevated temperatures and pressures in an autoclave (Henderson and Taylor, 1978; Weller and Wong, 1989; Vance et al., 2012; Cao et al., 2017; Chong et al., 2017; Nam et al., 2018). For fluorine, it is likely that captured fluoride byproducts could be discarded or immobilized in a fluoride-based waste form like a CaF_2_-based glass-ceramic waste form (Gregg et al., 2020).

## Phosphate Waste Forms

When formulating and synthesizing phosphate glasses containing high alkali contents, previous work can be drawn upon as well such as the work done with aluminophosphates, iron aluminophosphates, and iron phosphates where a range of phosphate precursors were used to produce glasses including P_2_O_5_, H_3_PO_4_, NaPO_3_, Al(PO_3_)_3_, AlPO_4_, and NH_4_H_2_PO_4_ (Brow, 1993; Brow et al., 1993; Day et al., 1998; Mesko et al., 2000; Siemer, 2012; Stefanovsky et al., 2014; Stefanovsky et al., 2017; Bai et al., 2021). In a study by Bai et al. (2021) directly comparing the same glass compositions produced with H_3_PO_4_ with those made using NH_4_H_2_PO_4_, glasses made with H_3_PO_4_ showed significantly higher Fe^3+^:Fe^2+^ ratios based on Mössbauer spectroscopy, which can lead to more chemically resistant (higher chemical durability) waste forms (Yu et al., 1997). This is another benefit for using H_3_PO_4_ as the phosphate additive when producing iron phosphate waste forms as opposed to ammonium hydrogen phosphates.

## Impacts on potential applications

Based on the information provided above, several potentially problematic species could be generated from reacting phosphates with halide salt streams. The main hazards include the corrosive hydrogen halides (i.e., HF, HCl, HBr, and HI) and shock-sensitive trihalide compounds (i.e., NCl_3_, NBr_3_, NI_3_, and NH_4_I_3_). As previously discussed, the thermal stabilities of all possible byproducts vary extensively meaning that processes and facilities for managing these byproducts will likely differ for each halide-based salt. The phase-change (*T*_b_s) and decomposition temperatures reported here are for pure compounds. Thus, these temperatures do not represent the actual conditions expected where water is present as a byproduct that will result in dilution (and therefore adjustments to the phase-change temperatures) of the pure compounds. Also, several of these byproducts have very low *T*_b_s and, if produced, would likely be vented as gases. However, it seems that after careful selection of the phosphate precursor, reaction temperatures, reaction times, the molar ratios of the scavenging reactant cation to the total halide content (e.g., NH_4_^+^:Cl^−^, H^+^:Cl^−^), and byproduct management, several options exist for removing the halides from salt-based nuclear wastes containing fission-product (Riley et al., 2020; Riley et al., 2021a). Since the primary MSR designs currently under consideration are either chloride-based or fluoride-based, these types of processes are possible for recycling the valuable ^37^Cl from chloride-based MSRs or removing the fluorine from fluoride-based wastes so that more effective waste management options are available for storing the fission product cations, such as phosphate waste forms like glass, crystallized glass, or glass ceramics (Siemer, 2012; Zhang et al., 2013; Liu et al., 2019; Riley et al., 2019; Riley et al., 2020; Wang et al., 2020; Riley et al., 2021a; Riley et al., 2021b). Management of the halide-based byproducts for waste disposal depends on the specific halide distribution and isotopes present for each, which will likely include some iodine-based species. Separate potential waste form options exist for halide-based species including mineral synthesis from solutions (e.g., caustic scrubbers) (Bollinger et al., 2022) into crystalline matrices like apatite [e.g., Ca_5_(PO_4_)_3_F, Ca_5_(PO_4_)_3_Cl, Pb_10_(VO_4_)_6_I_2_] (Metcalfe and Donald, 2004; Donald et al., 2007; Metcalfe et al., 2008; Vance et al., 2012; Cao et al., 2017), spodiosite [e.g., Ca_2_(PO_4_)Cl] (Metcalfe et al., 2008; Vance et al., 2012), and sodalite [e.g., Na_8_(AlSiO_4_)_6_Cl, Na_8_(AlSiO_4_)_6_I_2_] (Strachan and Babad, 1979; Nakazawa et al., 1999; Vance et al., 2012; Lepry et al., 2013; Chong et al., 2020).

## Summary and conclusion

This paper provides a very brief overview of the types of reactions and byproducts that can be expected when reacting halide-containing nuclear salt wastes with phosphate precursors such as H_3_PO_4_, NH_4_H_2_PO_4_, and (NH_4_)_2_HPO_4_. While most of the byproducts are very manageable from these types of reactions, the primary concerns come from potential trihalides that can form under certain conditions including NCl_3(s)_, NBr_3(s)_, NI_3(s)_, and NH_4_I_3(s)_, which are all shock-sensitive contact explosives. While these are potential byproducts from the reactions described herein, other byproducts are also possible from such streams that do not pose these types of issues such as ammonium halides (e.g., NH_4_I) and dihalides [e.g., I_2(g)_]. Due to the inherent instabilities with most of the triiodides, is also likely that these complexes will undergo decomposition prior to condensing depending on the processing conditions. In any case, removing the condensate products of these reactions and transporting them towards a caustic scrubber or molten hydroxide scrubber should be an adequate method for neutralization and preventing downstream transport or unwanted release to the environment. Formation of the triiodides can be avoided if H_3_PO_4_ is used instead of the ammonium hydrogen phosphates. The hydrogen halide byproducts can be neutralized in a caustic scrubber.
