# Supplementary figures and images for: Dehalogenation reactions between halide salts and phosphate compounds
Source: Front Chem. 2022 Sep 7;10:976781. doi: 10.3389/fchem.2022.976781 (PMC9518719; doi:10.3389/fchem.2022.976781)

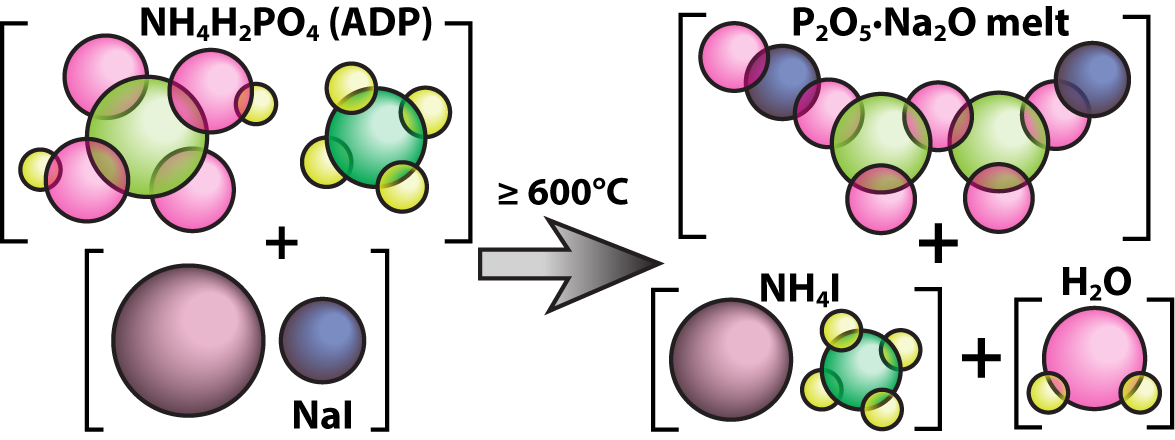

Supplement: Supplementary file 1 [file Image1.TIF]
